# Revolutionizing semen analysis: introducing Mojo AISA, the next-gen artificial intelligence microscopy

**DOI:** 10.3389/fcell.2023.1203708

**Published:** 2023-06-20

**Authors:** Pallav Sengupta, Sulagna Dutta, Shubhadeep Roychoudhury, Francesco Vizzarri, Petr Slama

**Affiliations:** ^1^ Physiology, Department of Biomedical Sciences, College of Medicine, Gulf Medical University, Ajman, United Arab Emirates; ^2^ School of Medical Sciences, Bharath Institute of Higher Education and Research (BIHER), Chennai, India; ^3^ Department of Life Science and Bioinformatics, Assam University, Silchar, India; ^4^ National Agricultural and Food Centre, Nitra, Slovakia; ^5^ Laboratory of Animal Immunology and Biotechnology, Department of Animal Morphology, Physiology and Genetics, Faculty of AgriSciences, Mendel University in Brno, Brno, Czechia

**Keywords:** computer-assisted semen analysis, male infertility, Mojo AISA, sperm, reproductive medicine

## 1 Introduction

### 1.1 Semen analysis: conventional methods *versus* Mojo AISA

Semen analysis is a critical diagnostic tool used to evaluate male fertility potential. The traditional method of semen analysis involves manual counting of sperm, which can be time-consuming and prone to inter-observer variability. (Jequier, 2010). Computer-assisted semen analysis (CASA) systems were developed to overcome these limitations and improve the accuracy and efficiency of semen analysis. (Krause, 1995). Recently, a new system called Mojo AISA has been introduced, which claims to offer superior performance compared to conventional CASA systems. (Parrella et al., 2022).

**FIGURE 1:**
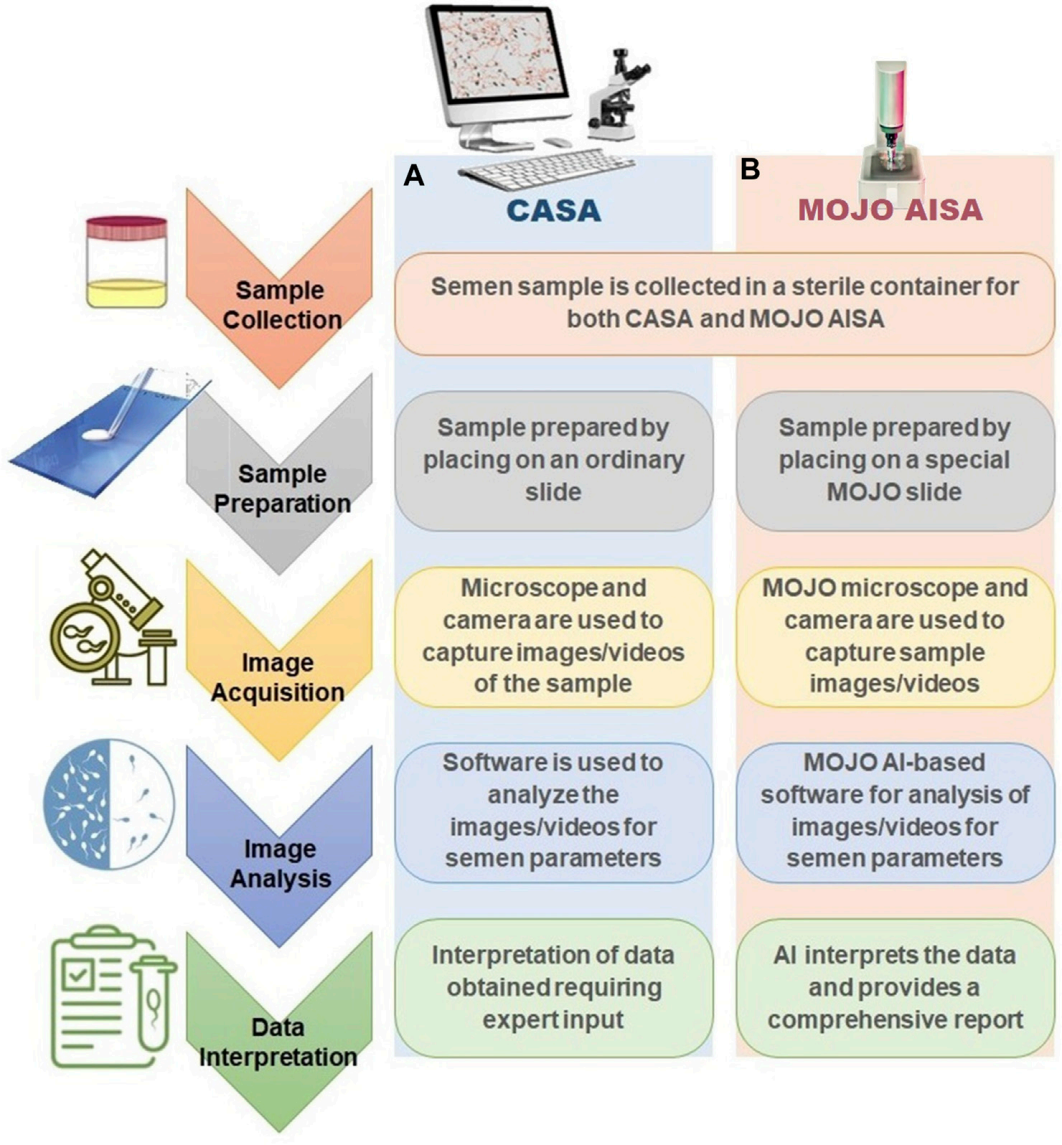
Computer-Assisted Semen Analysis (CASA) workstation and Mojo AISA (Automated Intelligent Sperm Analysis) protocol. **(A)** Traditional CASA workstation, comprising of a microscope, camera, and computer software for image analysis. This setup requires manual preparation of sperm samples and manual adjustments to the microscope, resulting in longer preparation time and potential operator-dependent errors. **(B)** Mojo AISA protocol, featuring its AI-driven process. This advanced method reduces manual intervention, as it integrates sample preparation, imaging, and analysis into a single automated process. Mojo AISA’s protocol employs machine learning algorithms to identify and assess sperm parameters with enhanced accuracy and speed.

Conventional methods of semen analysis typically involve assessing several parameters such as sperm concentration, motility, morphology, and vitality. (Jequier, 2010). These parameters are usually evaluated using various staining techniques, such as eosin-nigrosin, hematoxylin-eosin, and Diff-Quik. (Barbăroșie et al., 2021). The analysis is usually performed under a microscope, and the observations are recorded manually. The accuracy of these methods can be affected by inter-observer variability and subjective interpretation (Barbăroșie et al., 2021). In contrast, CASA systems use high-speed cameras and image analysis algorithms to automate the process of semen analysis (Figure 1) (Krause, 1995). The system can capture and analyze thousands of sperm in a matter of seconds, providing objective and accurate measurements of sperm parameters such as concentration, motility, velocity, and morphology. CASA systems use advanced algorithms to track the sperm movement and generate a range of statistics, such as curvilinear velocity, straight-line velocity, and average path velocity. The results are presented in a detailed report, providing clinicians with valuable information for diagnosis and treatment (Krause, 1995).

Mojo AISA is a new system that claims to offer several advantages over conventional CASA systems. It uses artificial intelligence and deep learning algorithms to analyze semen samples quickly and accurately (Figure 1) (Parrella et al., 2022). Mojo AISA can perform multiple analyses simultaneously, providing a comprehensive evaluation of sperm quality in a single test. The system can also detect subtle abnormalities in sperm morphology and flag them for further investigation. (Parrella et al., 2022) Mojo AISA is a newer system that claims to offer superior performance compared to conventional CASA systems, with the use of artificial intelligence and deep learning algorithms. The use of high-end scientific language is essential in describing the technical details and capabilities of these methods accurately (Parrella et al., 2022).

## 2 A study on Mojo AISA^3^: under the scrutiny glasses

The study aimed to assess the accuracy and reliability of Mojo AISA (Parrella et al., 2022), an Artificial Intelligence Semen Analysis system, in providing precise semen analysis results for daily routine use. The study is crucial because it provides evidence that the Mojo AISA technology has the potential to improve the accuracy and efficiency of semen analysis, leading to better diagnostic outcomes for male infertility. Thus, the present commentary on this pioneering study aims to concisely discuss the merits and limitation of Mojo AISA that can be inferred from the study outcome, and thereby to highlight the potential for artificial intelligence and machine learning algorithms to transform the field of reproductive medicine, paving the way for more innovative and effective diagnostic tools and treatments.

Currently, the most widely used method for performing semen analysis is through manual microscopy and/or CASA. However, the limitations of the classic image processing algorithms in discriminating sperm heads from other cells with similar size have led to improper results. In an attempt to surmount these limitations, Mojo AISA has been developed to accurately determine concentration and motility, utilizing a neural network classification system comprising an intricate series of embedded algorithms.

The above mentioned study assessed 64 semen samples from 62 men, over a 9-month period, employing both manual microscopy methods and Mojo AISA, adhering to the World Health Organization’s 5th Edition guidelines (Parrella et al., 2022). Concentration and motility parameters were duly evaluated and juxtaposed between the two methodologies, with a focus on Progressive (PR), Non-Progressive (NP), and combined motility (PR + NP) categories. Both normal and abnormal semen samples were incorporated in the study. Results showed that Mojo AISA was able to provide precise semen analysis results in a 50% shorter time compared to the manual method. This finding is significant as it minimizes the time required for embryologists to perform the procedure, thereby improving productivity and efficiency. Nonetheless, certain caveats warrant cautious interpretation of the results. One of the limitations of Mojo AISA is that it may present difficulty in assessing samples with extremely low concentration. Further evaluation is needed for this type of sample. Moreover, the protocol for preparing slides must be followed correctly to avoid the formation of air bubbles, which may impact the correct semen evaluation of Mojo AISA, misleading sperm results. The findings of the study have wider implications for the field of reproductive medicine as they suggest that Mojo AISA can provide more precise semen analysis results, with lower inter-laboratory variability, and in a shorter time. This may lead to an increased adoption of Mojo AISA in clinics and laboratories, thereby improving the accuracy and reliability of semen analysis results.

## 3 Conclusion

The article provides evidence that Mojo AISA can minimize human error and improve the objectivity of the semen analysis procedure by means of precise results. The concise discussion also shows that Mojo AISA can significantly reduce the time required to perform the procedure, which may lead to increased productivity and efficiency in embryology laboratories. Although limitations exist, Mojo AISA has the potential to improve the accuracy and reliability of semen analysis results, with wider implications for the field of reproductive medicine.
